# Phytochemical Characterization and Antimicrobial Activity of Several *Allium* Extracts

**DOI:** 10.3390/molecules28103980

**Published:** 2023-05-09

**Authors:** Ioana Andreea Barbu, Alexandra Ciorîță, Rahela Carpa, Augustin Catalin Moț, Anca Butiuc-Keul, Marcel Pârvu

**Affiliations:** 1 Faculty of Biology and Geology, Babeș-Bolyai University, 1 M. Koganiceanu Str., 400084 Cluj-Napoca, Romania; ioana.barbu@ubbcluj.ro (I.A.B.); alexandra.ciorita@ubbcluj.ro (A.C.); anca.keul@ubbcluj.ro (A.B.-K.); marcel.parvu@ubbcluj.ro (M.P.); 2 Doctoral School of Integrative Biology, Babeș-Bolyai University, 400015 Cluj-Napoca, Romania; 3 Center for Systems Biology, Biodiversity and Bioresources, Babeş-Bolyai University, Clinicilor Str., 400006 Cluj-Napoca, Romania; 4 National Institute for Research and Development of Isotopic and Molecular Technologies, 400293 Cluj-Napoca, Romania; 5 Institute for Research-Development-Innovation in Applied Natural Sciences, Babes-Bolyai University, 30 Fântânele Str., 400294 Cluj-Napoca, Romania; 6 Faculty of Chemistry and Chemical Engineering, Babes-Bolyai University, 11 Arany Janos Str., 400028 Cluj-Napoca, Romania; augustin.mot@ubbcluj.ro

**Keywords:** *Allium*, antimicrobial activity, allicin, phytochemical analysis

## Abstract

Microbial infections affect both the human population and animals. The appearance of more and more microbial strains resistant to classical treatments led to the need to develop new treatments. *Allium* plants are known for their antimicrobial properties due to their high content of thiosulfinates, especially allicin, polyphenols or flavonoids. The hydroalcoholic extracts of six *Allium* species obtained by cold percolation were analyzed regarding their phytochemical compounds and antimicrobial activity. Among the six extracts, *Allium sativum* L. and *Allium ursinum* L. have similar contents of thiosulfinates (approx. 300 μg allicin equivalents/g), and the contents of polyphenols and flavonoids were different between the tested species. The HPLC-DAD method was used to detail the phytochemical composition of species rich in thiosulfinates. *A. sativum* is richer in allicin (280 μg/g) than *A. ursinum* (130 μg/g). The antimicrobial activity of *A. sativum* and *A. ursinum* extracts against *Escherichia coli*, *Staphylococcus aureus*, *Candida albicans* and *Candida parapsilosis* can be correlated with the presence of large amounts of thiosulfinates. Both extracts have shown results against *Candida* species (inhibition zones of 20–35 mm) and against Gram-positive bacteria, *Staphylococcus aureus* (inhibition zones of 15–25 mm). These results demonstrate the antimicrobial effect of the extracts and suggest their use as an adjuvant treatment for microbial infections.

## 1. Introduction

The uncontrolled use of antibiotics in treatment and antimicrobial chemical preservatives in food preservation is the major cause of the emergence of antibiotic resistance [1,2]. Since the identification of microorganisms that cause various infections is not rigorously carried out, broad-spectrum antibiotics are used to treat these conditions, most of the time unnecessarily. This has led to the difficult treatment of some infections associated with the emergence of resistance to antibiotics that affect both the human population and various animals [3,4]. The emergence of multiresistant bacteria to antimicrobial agents and the ineffectiveness of common antibiotic treatments are increasing at an alarming rate. Among the multiresistant bacteria that affect animals are *Salmonella* spp., *S. aureus*, *Campylobacter* spp. and *E. coli*, which cause urinary, respiratory and reproductive tract infections [3]. This prompts researchers to look for new ways to fight microbial infections.

Plants can synthesize several active compounds that can have antibacterial [5], antifungal [6,7], antiparasitic [8] and antitumor effects [9], making them perfect candidates for the development of new antimicrobial agents.

Plants of the *Allium* genus are perennial and herbaceous and belong to the *Liliaceae* family. They have been known for their antimicrobial properties since ancient times, due to the presence of allicin in large amounts in their chemical composition [10,11,12].

In recent years, many studies have shown that garlic and its bioactive constituents (polyphenols, flavonoids and thiosulfinates), especially allicin, exhibit antioxidant, anti-inflammatory, antibacterial, antifungal and antitumor properties [13]. Plants of the *Allium* genus can also be used in the treatment and prevention of hypertension. Due to garlic’s anti-inflammatory properties, it could treat neurodegenerative diseases such as Alzheimer disease [14]. Extracts from these plants can have an important role in the adjuvant treatment of various microbial infections, due to the bioactive substances they contain. That is why the polarity of the solvent used to obtain the extracts influences their content, with the ethanolic extracts being richer in polyphenols and flavonoids than the methanolic or aqueous ones [15,16]. Garlics and onions have antibacterial compounds with effects against Gram-positive (*S. aureus*, *S.* spp.) and Gram-negative bacteria, (*Escherichia coli*, *Salmonella* spp., *Klebsiella* spp., *Pseudomonas aeruginosa* and *Helicobacter pylori*) [17,18,19,20,21]. Several studies demonstrate that *A*. *ursinum* can be used as an antimicrobial agent. Extracts prepared from its fresh flowers and leaves exert antifungal and antibacterial activities, directed predominantly on Gram-positive bacteria and less on Gram-negative. The antimicrobial potential of this plant is attributed to sulfur compounds, especially allicin [22].

The aim of this study is the analysis of the chemical composition of the hydroalcoholic extracts of two forms of *Allium cepa* L.—the Arieș red cultivar of *Allium cepa* and white variety of *A. cepa*, which were not previously investigated—and other common species: *Allium sativum* L., *Allium ursinum* L., *Allium fistulosum* L. and *Allium senescens* L. subsp. *montanum* (F. W. Schmidt) Holub. Investigating the antimicrobial activity of these extracts against *Escherichia coli*, *Staphylococcus aureus*, *Candida albicans* and *Candida parapsilosis*, common microorganisms involved in human and animal infections, was also one of our objectives. 

## 2. Results

### 2.1. Phytochemical Composition of Allium Extracts 

The total content of polyphenols, flavonoids and thiosulfinates in the *Allium* extracts is shown in Table 1. The total flavonoids content proved to be double in the extract of *A. sativum* compared to that of *A. ursinum*. The highest concentration of flavonoids was in the extracts of *A. senescens* subsp. *montanum* (F. W. Schmidt) Holub (52.6 ± 1.2 μg QE/g) and the Arieș red cultivar of *Allium cepa* (54.9 ± 2.4 μg QE/g), and the lowest in the extract of the white variety of *A. cepa* (18.6 ± 1.5 μg QE/g). The total polyphenols content was similar in the case of the extracts of *Allium senescens* L. subsp. *montanum* (F. W. Schmidt) Holub and the Arieș red cultivar of *Allium cepa*, which had the highest concentrations (423 ± 35 μg GAE/g and 418 ± 7 μg GAE/g). *A. fistulosum* and *A. ursinum* also had similar contents, and the lowest concentration was in the extract of the white variety of *A. cepa* (175 ± 11 μg GAE/g). The extracts of *A. sativum* and *A. ursinum* had a high content of thiosulfinates (333 ± 5 μg AE/g and 312 ± 7 μg AE/g, respectively). 

**Figure 1:**
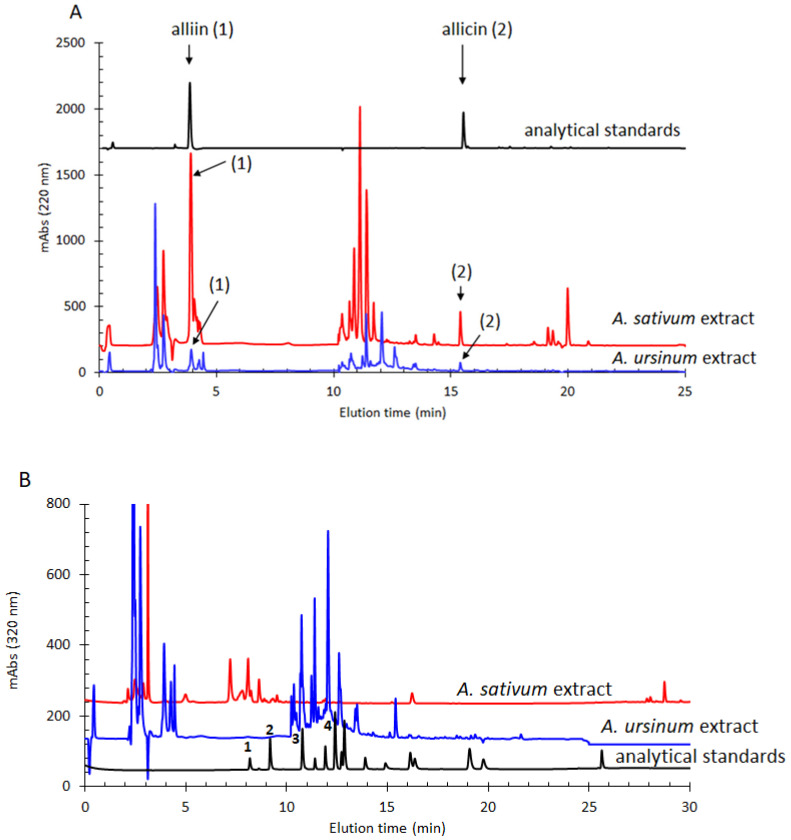
(**A**) Chromatographic profiles for alliin and allicin evaluation (220 nm) in the *A. sativum* and *A. ursinum* extracts. (**B**) Chromatographic profiles for phenolics evaluation (320 nm) in the *A. sativum* and *A. ursinum* extracts. Analytes determined: 1—gentisic acid, 2—chlorogenic acid, 3—4-hydroxybenzoic acid, 4—*p*-coumaric acid. For clarity, only the detected analytes are indicated in this figure. The other analytes used in this chromatographic method that were not detected in the analyzed sample are indicated in the Supplementary Materials (Table S1).

Using the HPLC-DAD method, the most important phenolic and thiosulfinate compounds present in the *A. sativum* and *A. ursinum* extracts that showed antimicrobial activity were determined. The two extracts showed different levels of alliin and allicin, with *A. sativum* being richer in these compounds than *A. ursinum*, as shown in Figure 1. Chlorogenic acid was present in a similar amount in the two extracts, while *p*-coumaric acid was found in a significantly higher amount in the *A. ursinum* extract (102 ± 10 μg/g). Gentisic acid and 4-hydroxybenzoic acid were evident in notable quantities only in the extract of *A. sativum*, as can be seen in Table 2.

### 2.2. Antimicrobial Activity

**Figure 2:**
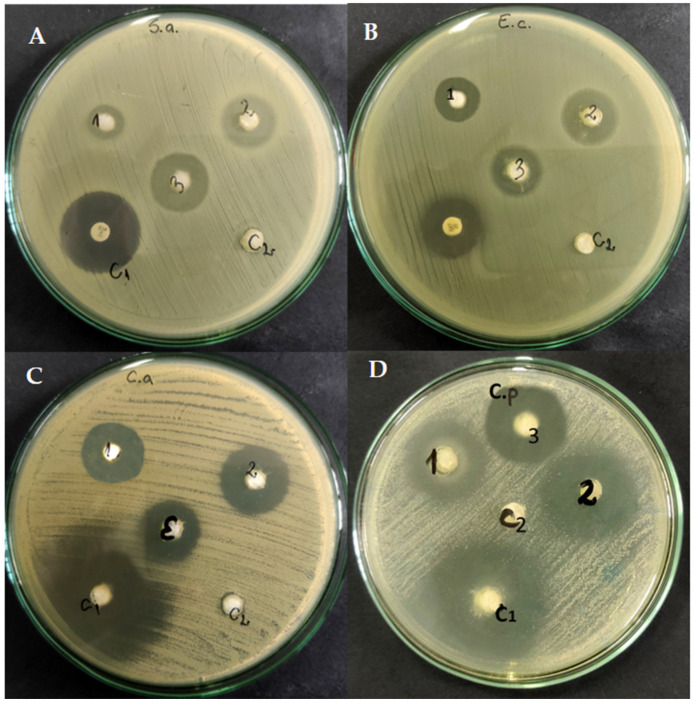
Antimicrobial activity of *Allium sativum* (1), *A. ursinum* (2), *A. sativum* and *A. ursinum* 1:1 extracts (3), Ciprofloxacin/Sulfamethoxazole/Fluconazole (c1AC1/c1B/c1C,D) and ethanol 30% (c2) on *S. aureus* (**A**), *E. coli* (**B**) *C. albicans* (**C**) and *C. parapsilosis* (**D**).

**Figure 3:**
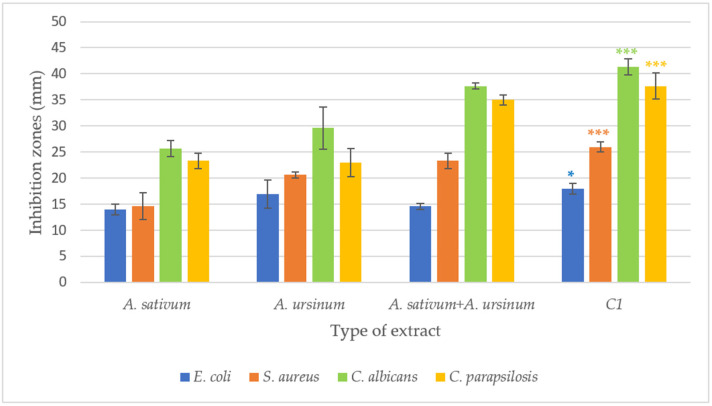
The antimicrobial effect of *Allium sativum*, *A. ursinum*, *A. sativum* and *A. ursinum* 1:1 on *S. aureus E. coli*, *C. albicans* and *C. parapsilosis*. The values represent the mean of three measurements ± standard deviation; *** *p* < 0.0005, * *p* < 0.05, according to one-way ANOVA.

Out of the six *Allium* extracts tested, only two showed antimicrobial effects: *A. sativum* and *A. ursinum* (Figure S1). Both extracts were more effective against *Candida* species compared to bacterial ones. *S. aureus* proved to be more sensitive to the tested extracts than *E. coli* (zones of inhibition of 15–22 mm vs. 15–20 mm)*. A. ursinum* extract compared to that of *A. sativum* had larger inhibition zones against *S. aureus* than *E. coli* (Figure S1 and Figure 2). For the fungal species tested, the extract of *A. ursinum* (inhibition zones of 20–32 mm) was more effective than that of *A. sativum* (inhibition zones of 22–27 mm) (Figure 2). The effects against *C. albicans* and *C. parapsilosis* were more promising compared to the bacteria tested. At the same time, the 1:1 combination of the two extracts was also tested (Figure 2 and Figure 3). For *E. coli*, no differences were observed between the combination of extracts and the individual extracts. In the case of *S. aureus*, a slight increase in the inhibition zone (22–25 mm) was observed, and for *Candida* strains, an increase in the inhibition zones was observed compared to the individually tested extracts. For all microorganisms tested, the diameters of the inhibition zones of the antibiotics and antifungals used are close to those of the extracts. The 1:1 combination of the two extracts, tested on *C. parapsilosis*, had an effect close to that of Fluconazole (32 mm vs. 36 mm). For the agar diffusion method, three independent experiments were conducted and the mean was calculated (Figure 3). According to one-way ANOVA statistical analysis, all values are statistically significant at *p* value < 0.05, as compared to the control. 

The microdilution method confirmed the results obtained by antibiograms and antifungigrams using the diffusion method (Table 3). The extracts of *A. sativum* and *A. ursinum* had antimicrobial effects on the strains *E. coli* ATCC 25922 and *S. aureus* ATCC 25923 and two *Candida* species: *C. albicans* ATCC 10231, *C. parapsilosis* ATCC 22019. The 1:1 combination proved to be more effective than the 1:2 (*A. sativum*:*A. ursinum*) and 2:1 (*A. ursinum*:*A. sativum*) combinations.

The antimicrobial activity of *A. sativum*, *A. ursinum*, and mixed *A. sativum* and *A. ursinum* 1:1 extracts was tested on four microbial strains. In addition, for each strain, we also used a control of 30% ethanol (C2) and a control of an antimicrobial substance (C1): ciprofloxacin for *S. aureus*, sulfamethoxazole for *E. coli* and fluconazole for *Candida* species. Compared to the control (C1 = antimicrobial substances), the antimicrobial activity of *Allium* extracts was good. For some experimental variants, it exhibits an inhibition of almost half of the inhibition value given by the control substance and for other experimental variants it reaches almost the level of inhibition of the chosen antimicrobial substance (Figure 3).

In these experiments, the bacteriostatic (MIC) and bactericidal effects (MBC) of *Allium* extracts against *E. coli*, *S. aureus*, *C. albicans* and *C. parapsilosis* were also determined.

The minimum inhibitory concentration (MIC) of the *A. sativum* and *A. ursinum* extracts tested on bacteria and fungi varied depending on the species and the plant extract (Table 3). It can be observed that the MIC of the extracts is higher for Gram-positive bacteria (12.5) compared to Gram-negative (6.25).

The minimum bactericidal concentration (MBC) was determined by diluting each well in tenfold dilutions. From each dilution, aliquots were transferred on agar plates and incubated for 24 h. The first concentration at which no colony forming units were observed was considered the MBC.

The MICs for the tested microbial species were similar to the MBCs for some *Allium* sp. extracts, but the tested microbial species generally showed a lower concentration for MBC (Table 3). There were also discrepancies between MICs and MBCs, e.g., for the, *A. ursinum* extract on *C. parapsilosis* (MIC = 6.25 and MBC = 25) and for the *A. sativum* extract on *E. coli* (MIC = 6.25 and MBC = 25).

## 3. Discussion

*Allium* plants have been known for their antimicrobial properties since ancient times. Recent studies have shown that the leaves and bulbs of these plants are rich in phenols, flavonoids and thiosulfinates, compounds that have proven antimicrobial, antioxidant or antitumor activity [23,24,25]. Naturally, in *Allium* bulbs or leaves allicin is formed after crushing or cutting the plant organs under the action of the allinase enzyme [24].

The chemical composition of the extracts depends on the species. In our study, we compared five *Allium* species, two of which are *A. cepa* varieties, which have not been previously analyzed, in order to correlate the phytochemical composition with the antimicrobial activity. Although the Arieș red cultivar of *Allium cepa* and *Allium senescens* L. subsp. *montanum* (F. W. Schmidt) Holub had the highest content of flavonoids and phenols, respectively, according to Table 1, *Allium sativum* and *Allium ursinum* had the highest content of thiosulfinates among the tested species. It is known that allicin is a compound with high antimicrobial activity, so we can correlate the antimicrobial activity of the two species with the presence of the high content of thiosulfinates [26]. Moreover, extracts of *A. sativum* and *A. ursinum* had a high content of polyphenols, indicating a synergistic effect of the two classes of compounds [5,25], which was observed in our study as well. In addition, the total content of polyphenols varies depending on the species, the organ of the plant from which the extract is obtained and its maturity. Plants in the flowering period have a higher polyphenol content than in the growing period, and the leaves are the richest in these compounds [27,28,29]. Nhut et al. (2020) also analyzed the phytochemical composition of an *Allium tuberosum Rottler* ex *Spreng* extract and their results were similar with ours regarding the total content of flavonoids (31.10 ± 1.12 mg QE/g) [30] but the total content of polyphenols is higher in the extracts used for this study. *Allium schoenoprasum* and *Allium vineale* also have a similar content of polyphenols, flavonoids and sulfur compounds to the tested extracts in our study [31,32]. The extract of *A. scorodoprasum* L. subsp. *rotundum* instead has a lower total content of polyphenols (25.60 ± 0.48 mg GAE/g) and flavonoids (2.21 ± 0.04 mg QE/g) than the extracts analyzed in this study [33]. In addition, the solvent used to obtain the extracts is an important factor that influences the content of polyphenols and flavonoids due to their different solubility [34]. It was proven that the ethanolic onion extract was the richest in polyphenols and flavonoids compared to the methanol, acetone or aqueous extracts [34,35].

The antimicrobial activity of *A. ursinum* and *A. sativum* extracts is also documented in the literature [26,36], and the results obtained are correlated with the already existing ones. The *A. ursinum* extract was more effective against Gram-positive than Gram-negative bacteria both in our study and in previous studies [5]. The antimicrobial properties of the *A. cepa* extract were previously examined against *S. aureus*, *E. coli*, *Bacillus cereus*, *Listeria innocua* and *Pseudomonas aeruginosa*. The extracts at different concentrations showed antibacterial activity against all tested bacterial strains. The extract at the highest concentration was more effective against *E. coli* and least effective against *P. aeruginosa* [20,21,37]. Although previous studies showed antimicrobial effects produced by extracts of *Allium cepa*, our results did not show antimicrobial activity. *Allium fistulosum* was also not effective against *Pseudomonas aeruginosa* at any concentrations tested [38]. This fact may be due to the method of obtaining the extracts and the solvents used. The *Allium sativum* petroleum ether extract was the most effective against *Staphylococcus* spp., followed by *E. coli* and *Proteus* spp. Likewise, the chloroform extract was more active against *Pseudomonas* spp. than the aqueous extract. The methanol and aqueous extracts presented some of the smallest zones of inhibition against the pathogens tested [39].

Due to the antimicrobial activity of the extracts of *A. sativum* and *A. ursinum*, we performed an analysis of extract combination to demonstrate their synergistic activity. The results obtained showed an additive effect of inhibiting the tested microorganisms but without a high increase in the inhibition zones. Moreover, the combination of extracts was more effective against *S. aureus* than *E. coli* and proved to be more effective against *Candida* spp., for which they also had larger inhibition zones individually. To our knowledge, the combination of these two extracts has not been tested yet but could be of interest for future research.

## 4. Materials and Methods

### 4.1. Plant Material and Extract Preparation

The plant extracts of *Allium sativum*, *Allium cepa* cv. Arieș red and the white variety of *Allium cepa* were obtained from bulbs collected from the private garden of Cluj-Napoca and kept in optimal conditions until November and December 2021. For *Allium fistulosum*, *Allium senescens* subsp. *montanum* and *Allium ursinum* extracts, the leaves collected in April 2022 and September 2021 from the “Alexandru Borza” Botanical Garden of Cluj-Napoca (46°45′36″ N and 23°35′13″ E) were used.

The plants were taxonomically identified and authenticated and voucher specimens (CL 666161 for *A. sativum*; CL 663978 for *Allium cepa* cv. Arieș red; CL 659761 for *A. fistulosum*; CL 659563 for *A. senescens* subsp. *montanum*; and CL 659750 for *A. ursinum*) were deposited in the Herbarium of “Alexandru Borza” Botanical Garden, “Babeș-Bolyai” University, Cluj-Napoca, Romania.

Fresh *Allium* plant material was washed with distilled water and cut into 1 cm fragments then put in the percolator, where it was extracted with 70% ethanol (Merck, Bucuresti, Romania) by cold repercolation method, at room temperature, for 3 days [40,41]. After filtration, the *Allium* extracts had a final concentration of 30% ethanol, except *A. cepa* cv. Arieș red, for which the ethanol concentration was 25% ethanol. The weight-to-volume ratios (*w*:*v* or g:mL) of the extracts were the following: 1:1 (*A. sativum*, *A. cepa* cv. Aries red, white variety of *A. cepa*), 1:1.1 (*A. senescens* subsp. *montanum*), 1:1.2 (*A. ursinum*) and 1:1.5 (*A. fistulosum*). The extraction yields were, thus, 1%, 1.1%, 1.2% and 1.5%, respectively.

### 4.2. Phytochemical Analyses of the Allium Extracts 

#### 4.2.1. Total Polyphenolic Content Procedure (TPC)

Before carrying out the protocols, the extracts were centrifuged for 5 min at 16,500 rpm.

The total content of polyphenols was determined using a Folin–Ciocâlteu reducing capacity assay: 50 µL of each extract together with 30 µL of Folin–Ciocâlteu reagent (Merck) were added to a volume of 1320 µL of distilled water in a PP 2 mL tube. A volume of 100 µL of 10% Na_2_CO_3_ was then added to this mixture. The mixture was incubated in the dark for one hour and then the absorbances were measured at 735 nm. Gallic acid (Merck) was used as the standard for the calibration curve with the following final concentrations: 50, 100, 150, 200, 300, 400 and 500 μg/mL (Figure S3). The TPC was calculated in each sample based on the calibration curve and was expressed as gallic acid equivalents (μg GAEs/g). The analysis was performed in triplicate and the results are expressed as mean ± standard error [42].

#### 4.2.2. Total Flavonoid Content Procedure (TFC)

The total flavonoid content (TFC) was determined by AlCl_3_ complexation. Thus, 250 µL of distilled water, 600 µL of 1 M sodium acetate pH 4.5, 300 µL of AlCl_3_ and 350 µL of each extract were mixed. After 20 min of incubation, the absorbances were measured at 452 nm. For the calibration curve (Figure S4), quercetin (Sigma) was used as the standard (final cuvette concentrations of 0, 3, 7, 10, 13, 20, 27 and 33 μg/mL). Using this calibration curve, the TFC was expressed as quercetin equivalents (μg QEs/g). The analysis was performed in triplicate and the results are expressed as mean ± standard error [43].

#### 4.2.3. Total Thiosulfinate Content Procedure (TTC)

To determine the total content of thiosulfinates, the 4-mercaptopyridine assay was used. A stock solution of 4-mercaptopyridine 90 mM in 70% ethanol was prepared. A volume of 125 µL of 4-mercaptopyridine stock solution was mixed in 50 mL of phosphate buffer pH 7.2, yielding a concentration of 0.225 mM of 4-mercaptopyridine. A volume of 950 µL from this buffer containing 4-mercaptopyridine was mixed with 50 µL of each extract. The solutions were incubated for 60 min and the 200–900 nm spectrum was measured at time intervals of 60, 75, 90, 105 and 120 min. The calculated analytical signal was the difference of the absorbances at time zero and after 120 min (A_0min_ − A_120min_) at 324 nm. A calibration curve (Figure S5) was constructed using a pure allicin standard (prepared as previously described by Pârvu et al., 2019) [44], using exactly the sample procedure. The concentrations of the allicin standards were as follows: 0, 5, 10, 20, 50, 100 and 150 μg/mL [45]. The analysis was performed in triplicate and the results are expressed as mean ± standard error [46]. 

### 4.3. Chromatographic Analysis of the Allium Extracts

The extracts that exhibited antimicrobial analysis, namely *A. sativum* and *A. ursinum*, have been analyzed in more detail using HPLC-DAD instrumentation. For this purpose, the extracts were filtered with a 0.22 μm filter. Two chromatographic procedures that were previously validated were used for the analysis of the two *Allium* extracts [44]. Briefly, the chromatographic analysis was performed on an Agilent 1200 HPLC instrument that was equipped with a quaternary pump and vacuum degasser. During the analysis, the extracts were kept in a temperature-controlled sample tray at 5 °C and were automatically injected into a Zorbax SB-C18 column (250 mm × 4.6 mm, 5 µm particle size, Agilent) that was held in a thermostat compartment at 30 °C. The detection was performed using a DAD detector. The flow rate was 1 mL/min and the injected sample volume was 15 µL. For the analysis of allicin and alliin (method 1), the protocol that was used employed 10 mM ammonium formate pH 2.5 as solvent A and acetonitrile as solvent B and a multistep gradient for elution as follows: isocratic at 0% B for 0–5 min, then the gradient increased linearly from 0 to 70% B for 5–14 min and from 70% to 90% B for 14–15 min and from 90% to 100% B for 15–18 min followed by an isocratic step at 100% B for 18–22 min and back to 0% B for 22–22.1 min, where it was kept for equilibration until 25 min. For this method, the chromatograms were monitored at 220 nm and the entire spectrum was measured every 2 s in the 210–260 nm region. Calibration curves were generated at six levels of concentration (39, 78, 156, 313, 625 and 1250 µg/mL) for both allicin (R^2^ = 0.9999) and alliin (R^2^ = 0.9994, Supplementary Material Figure S5) determination. For the analysis of phenolic acids, a distinct approach was optimized (method 2) that employed 10 mM ammonium acetate pH 5.5 as solvent A and acetonitrile as solvent B. For this method, the elution gradient was as follows: isocratic at 5% B for 0–2 min followed by a linear gradient from 5 to 35% B for 2–10 min, from 35% to 45% B for 10–20 min, from 45% to 95% B for 20–25 min, from 95% to 100% B for 25–28 min and an isocratic step at 100% B for 28–32 min, and then the column was re-equilibrated back to 5% B for 32–32.1 min and kept for complete re-equilibration until 35 min. For this second method, the chromatograms were monitored at 320 nm and the calibrations were conducted at seven levels of concentration (35, 53, 70, 105, 140, 210 and 280 µg/mL) for each standard (gentisic acid, chlorogenic acid, 4-hydroxybenzoic acid, *p*-coumaric acid, R^2^ > 0.9959, Supplementary Material Figure S6). The stock solution of the individual standard was created at 2 mg/mL in pure ethanol and diluted accordingly so that a mixture of the standards at the above-indicated concentration was obtained, also in pure ethanol. All the analyses were performed in duplicates and the results are expressed as mean ± standard error. The standards used for HPLC analysis were procured from commercial sources (detailed information in Supplementary Material Table S1 and Figure S7), except for allicin, which was synthesized and purified in our laboratory as previously described [40], and the purity was assessed by ^1^H NMR to at least 94% using the relative 100% method. Another allicin batch synthesis led to a purity of 95% after both ^1^H NMR and HPLC-MS assessment, thus supporting the reproducibility of allicin synthesis and purity analysis [47].

### 4.4. Disk Diffusion Method

The antimicrobial activity of the extracts was studied on *E. coli* ATCC 25922, *S. aureus* ATCC 25923 and two *Candida* species: *C. albicans* ATCC 10231 and *C. parapsilosis* ATCC 22019.

A Mueller–Hinton-agar (MH) medium was poured into Petri dishes. After solidification, 6 mm wells were created using a sterile cut tip. A suspension of pure culture microorganisms with a concentration of 0.5 on the MacFarland scale was inoculated onto these plates using a sterile equivion. A piece of sterile cotton with a diameter of 6 mm was placed in each well, over which 100 µL of each extract and 100 µL of 30% alcohol were pipetted. The plates were left to incubate for 24 h, at 37 °C, then the inhibition zones were measured [37,48]. The analysis was performed in triplicate and the results are expressed as mean ± standard error.

### 4.5. The Microdilution Method (MIC and MBC)

For the microdilution method, 96-well plates were prepared. An MH medium (100 µL) was inoculated into each well. In the first row of wells, a 100 µL extract was loaded, and for the following ones, serial dilutions were made with concentrations of 50, 25, 12.5 and 6.25% extract in the medium. The microbial suspension of 0.5 MacFarland turbidity was inoculated into each well (20 µL). The plates were incubated for 24 h at 35 °C, then the absorbances were read at 600 nm, using the BioTech Epoch plate reader by BioTek Instruments, Winooski, VT, USA and Gen5 Software (version 1.09) [38,49]. The MIC dilution wells were without visible growth and the control wells were subcultured on culture media and incubated at 37 °C, then colonies were counted after 12 and 24 h. Organisms grown from the control wells were compared to those grown from MIC test wells without visible growth. The same concentrations were used to determine the minimum bactericidal concentration (MBC). The lowest concentration of *Allium* species that killed 99% of the bacteria was considered the minimum bactericidal concentration (MBC) [50,51].

### 4.6. Statistical Analyses

Each analysis and antimicrobial activity was performed in triplicate, and the mean and standard deviation were then calculated. For our experiments, one-way ANOVA statistical analyses was performed using a free site (https://goodcalculators.com/one-way-anova-calculator/, accessed on 3 May 2023). Values of *p* ≤ 0.05 were considered statistically significant. 

## 5. Conclusions

The chemical compositions of *A. sativum*, *A. ursinum*, the white variety of *A. cepa*, the Arieș red cultivar of *A. cepa*, *A. fistulosum* and *A. senescens* subsp. *montanum* differ depending on the species; this fact also influences their antimicrobial activity. It was observed that the species with the highest contents of thiosulfinates also have the best antimicrobial activity against *E. coli*, *S. aureus*, *C. albicans* and *C. parapsilosis*. Moreover, *A. sativum* and *A. ursinum* had a high content of polyphenols, which potentiated their antimicrobial effect. Although these two species had effects against the bacteria and fungi tested, their combination had an additive effect, without highly increasing the zones of inhibition.

Due to their antimicrobial activity, these extracts could be used as adjuvant treatments for microbial infections or for the development of new drugs and could be further tested in combination with other compounds against various pathogens infecting humans and animals.

## Figures and Tables

**Table 1:** Phytochemical compounds of the analyzed *Allium* species. The values are indicated as μg analyte per g of wet plant material.

Species	TFC (μg QE/g)	TPC (μg GAE/g)	TTC (μg AE/g)
*A. sativum*	44.5± 4.1	278 ± 15	333 ± 5
*A. senescens L. subsp. montanum*	52.6 ± 1.2	423 ± 35	21 ± 2
*A. fistulosum*	27.6 ± 0.3	327 ± 8	36 ± 2
*A. cepa* cv. Arieș red	54.9 ± 2.4	418 ± 7	12 ± 4
White variety of *A. cepa*	18.6 ± 1.5	175 ± 11	5 ± 1
*A. ursinum*	22.9 ± 0.2	354 ± 13	312 ± 7

TFC—total flavonoid content (QEs—quercetin equivalents); TPC—total phenolic content (GAEs—gallic acid equivalents); TTC—total thiosulfinates content (AEs—allicin equivalents).

**Table 2:** Phytochemical analysis (HPLC-DAD) for the identified analytes in the two analyzed *Allium* species. The values are indicated as μg analyte per g of wet plant material.

Analyte (μg/g)	*A. sativum*	*A. ursinum*
Gentisic acid	38 ± 5	<LOD
Chlorogenic acid	36 ± 3	40 ± 5
4-hydroxybenzoic acid	16 ± 3	<LOD
*p*-coumaric acid	26 ± 4	102 ± 10
Alliin	1580 ± 30	260 ± 15
Allicin	280 + 15	130 ± 10

**Table 3:** The minimum inhibitory concentration (MIC) and minimum bactericidal concentration (MBC) (%) of *Allium* extracts.

Extracts	*E. coli*		*S. aureus*		*C. albicans*		*C. parapsilosis*	
	MIC	MBC	MIC	MBC	MIC	MBC	MIC	MBC
*A. sativum*	6.25	25	12.5	25	12.5	12.5	6.25	12.5
*A. ursinum*	6.25	12.5	25	50	6.25	12.5	6.25	25
*A. sativum* + *A. ursinum* 1:1	6.25	12.5	6.25	12.5	6.25	12.5	6.25	12.5
*A. sativum* + *A. ursinum* 1:2	6.25	12.5	12.5	12.5	50	50	6.25	12.5
*A. sativum* + *A. ursinum* 2:1	50	50	50	50	12.5	25	50	50

## Data Availability

Not applicable.

## Supplementary Materials

The following supporting information can be downloaded at: https://www.mdpi.com/article/10.3390/molecules28103980/s1, Figure S1. Antimicrobial activity of (1) *A. cepa* “*Roșie de Arieș*”, (2) *A. cepa* var. *alba*, (3) *A. senescens subsp. montanum* (F. W. Schmidt) Holub, (4) *A. sativum*, (5) *A. fistulosum*, C1 = ethanol 30%, C2 = ethanol 25% against (A) *C. albicans*, (B) *C. parapsilosis* (C) *E. coli*, (D) *S. aureus.* Figure S2. UV-vis molecular absorbance spectra of the standard solution and the calibration curve used for TPC determination, according to the described protocol. Figure S3. UV-vis molecular absorbance spectra of the standard solution and the calibration curve used for TFC determination, according to the described protocol. Figure S4. Time-dependence UV-vis molecular absorbance spectra for a standard solution and A. sativum sample and the calibration curve used for TTC determination, according to the described protocol. Figure S5. Calibration curve for allicin and alliin determination. Figure S6. Calibration curve for phenolic acids determination. Table S1. Supplementary information and classification for the analytical standards used in this study. The chromatograms of all the standards at different wavelength are indicated in Figure S7. Figure S7. Chromatograms monitored at different wavelengths for all the polyphenolic analytes that assessed in samples. Only four phenolic acids were detected in the analysed samples and are indicated with blue colour (here numbers 1, 3, 5 and 9 and their corresponding name is in Table S1).
