# Supplementary material for: Phytochemical Characterization and Antimicrobial Activity of Several Allium Extracts
Source: Molecules. 2023 May 9;28(10):3980. doi: 10.3390/molecules28103980 (PMC10222200; doi:10.3390/molecules28103980)
Supplement: Supplementary file 1 [file molecules-28-03980-s001.zip › molecules-2325308-supplementary.pdf]

Supplementary material

Title of the manuscript: ***Phytochemical Characterization and Antimicrobial Activity of Several Allium Extracts***

Authors: Ioana Andreea Barbu, Alexandra Ciorîță, Rahela Carpa, Augustin Catalin Moț, Anca Butiuc-Keul and Marcel Pârvu

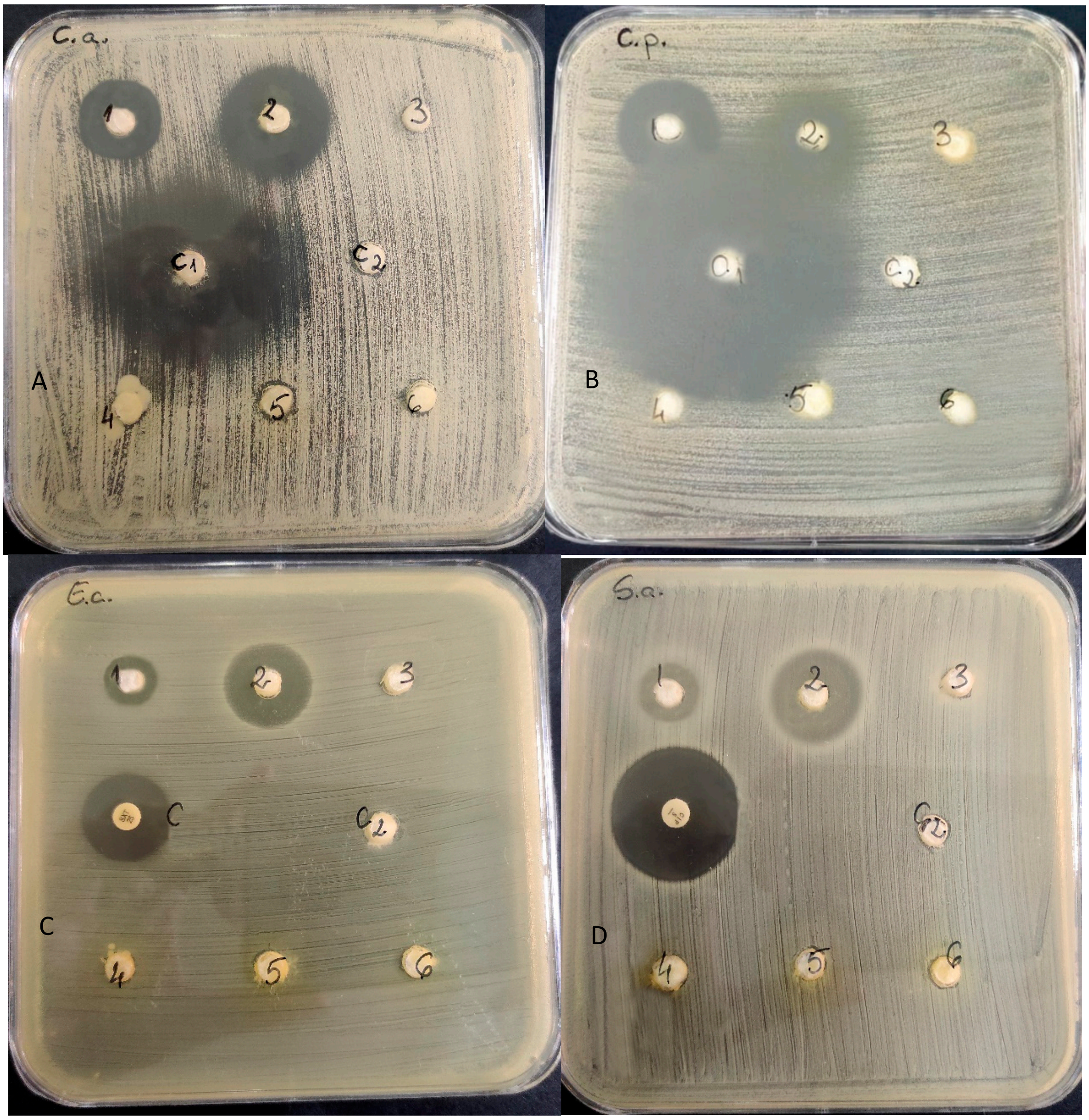

Figure S1. Antimicrobial activity of (1) *A. sativum*, (2) A. ursinum (3) white variety of *Allium cepa*, (4) Arieș red cultivar of *Allium cepa*, (5) *A. senescens subsp. montanum* (F. W. Schmidt) Holub, (6) *A. fistulosum*, C2= Ciprofloxacin/Sulfamethoxazole/Fluconazole (c1C/c1D/c1A,B) C2=ethanol 30%, C2=ethanol 25% against (A) *C. albicans*, (B) *C. parapsilosis* (C) *E. coli*, (D) *S. aureus*.

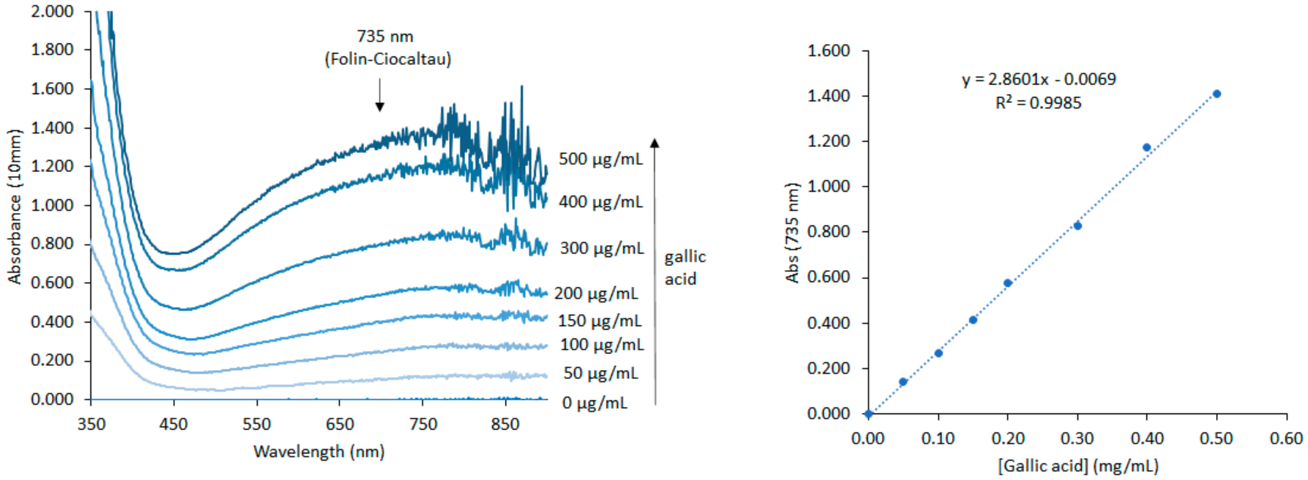

Figure S2. UV-vis molecular absorbance spectra of the standard solution and the calibration curve used for TPC determination, according to the described protocol.

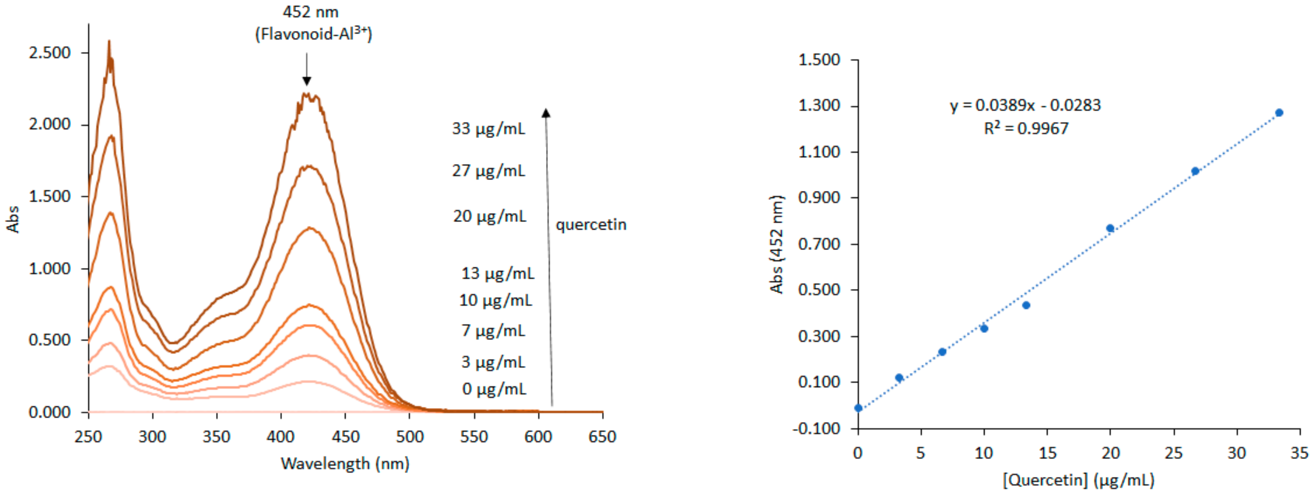

Figure S3. UV-vis molecular absorbance spectra of the standard solution and the calibration curve used for TFC determination, according to the described protocol.

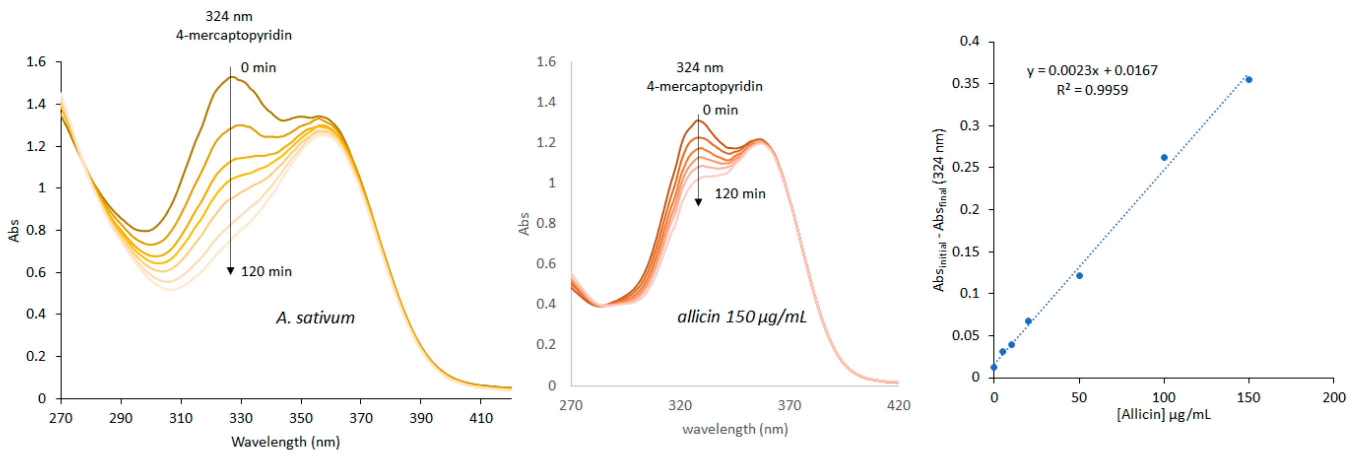

Figure S4. Time-dependence UV-vis molecular absorbance spectra for a standard solution and A. sativum sample and the calibration curve used for TTC determination, according to the described protocol.

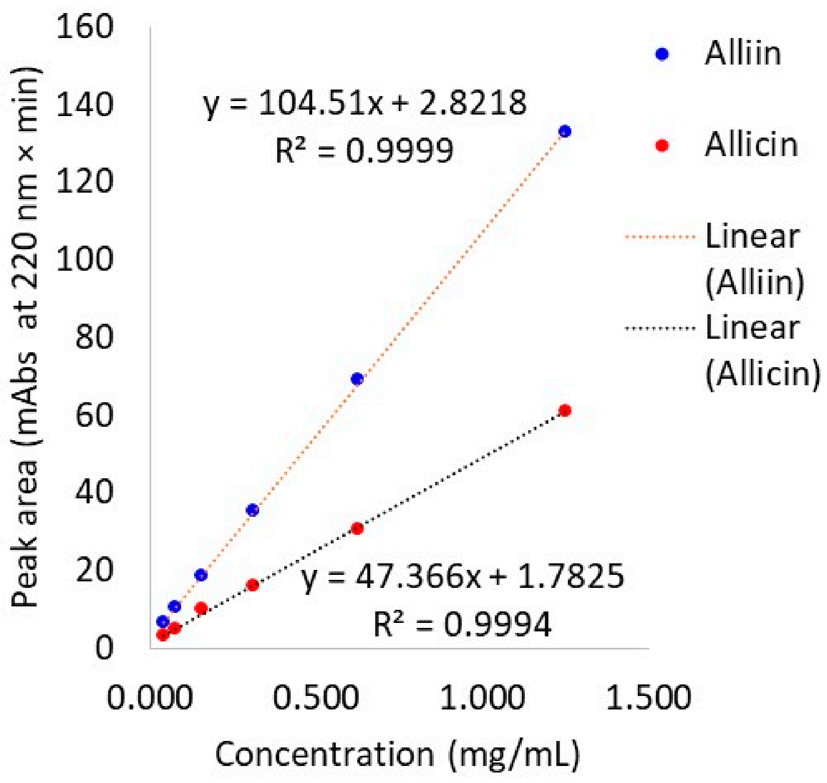

Figure S5. Calibration curve for allicin and alliin determination.

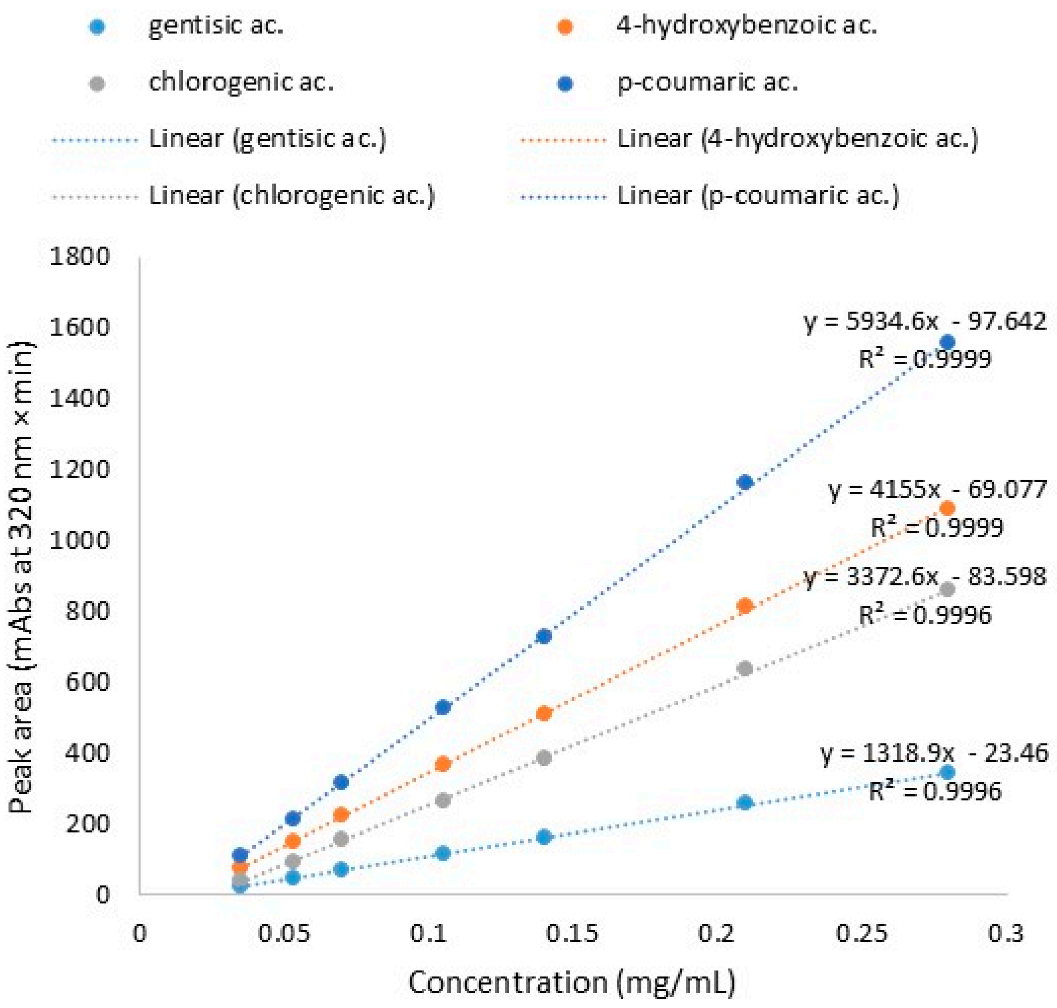

Figure S6. Calibration curve for phenolic acids determination.

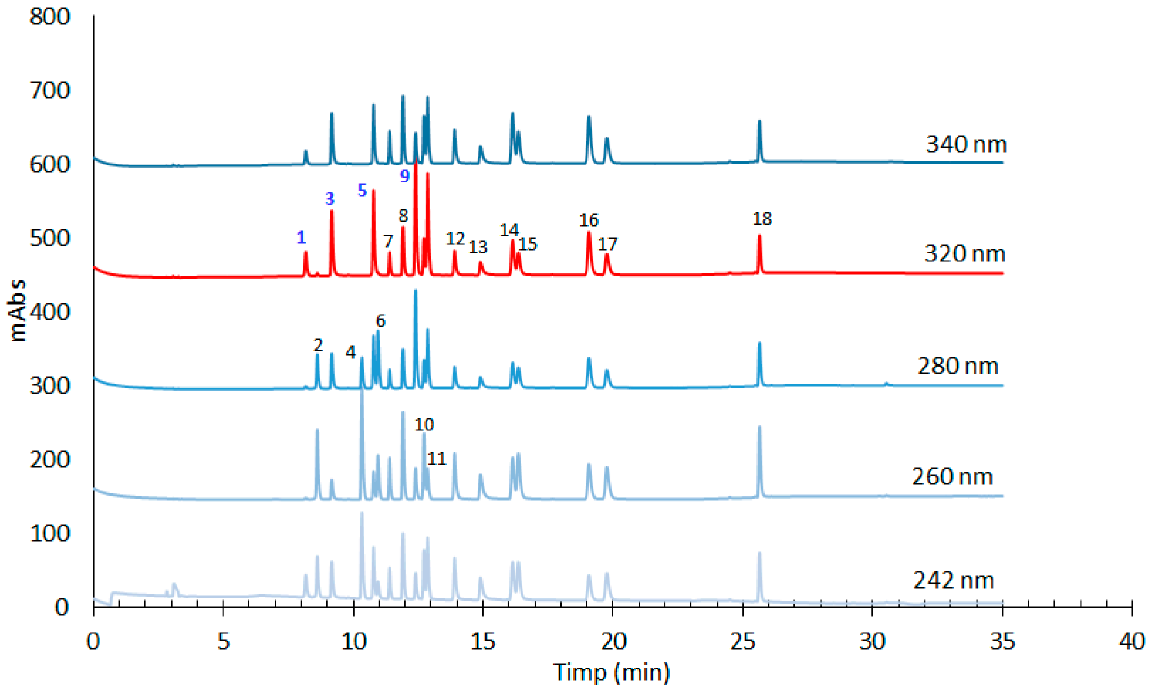

Figure S7. Chromatograms monitored at different wavelengths for all the polyphenolic analytes that assessed in samples. Only four phenolic acids were detected in the analysed samples and are indicated with blue colour (here numbers 1, 3, 5 and 9 and their corresponding name is in Table S1).

Table S1. Supplementary information and classification for the analytical standards used in this study. The chromatograms of the polyphenolic standards at different wavelength are indicated in Figure S7.

No.	Standard	Phytoconstituent class	Chromatographic method[a]	Retention time (min)	Detected in the analysed samples?	Producer, cod standard	Purity (%)
1	allicin	thiosulfinate	M1	3.68	**yes**	*in-house* synthesized	≥94[b]
2	alliin	sulfoxide	M1	15.42	**yes**	Sigma, 74264	≥90
1	gentisic ac.	phenolic acid	M2	8.13	**yes**	Sigma, 149357	≥98
2	3,4-dihydroxybenzoic	phenolic acid	M2	8.58	no	Sigma, 37580	≥97
3	Chlorogenic ac.	phenolic acid	M2	9.15	**yes**	Sigma, C3878	≥95
4	Caffeic ac.	phenolic acid	M2	10.76	no	Sigma, C0625	≥98
5	4-hydroxybenzoic	phenolic acid	M2	10.31	**yes**	Sigma, H20059	≥99
6	Syringic ac.	phenolic acid	M2	10.94	no	Sigma, S6881	≥95
7	Rutin	flavonoid	M2	11.39	no	Sigma, R2303	≥95
8	Isoquercitrin	flavonoid	M2	11.91	no	Sigma, 17793	≥90
9	p-coumaric	phenolic acid	M2	12.38	**yes**	Sigma, C9008	≥98
10	Quercitrin	flavonoid	M2	12.72	no	Sigma, 00740580	not specified
11	Ferulic ac.	phenolic acid	M2	12.85	no	Sigma, 128708	≥99
12	Myricetin	flavonoid	M2	13.89	no	Sigma, M6760	≥96
13	Morin	flavonoid	M2	14.88	no	Sigma, M4008	not specified
14	Luteolin	flavonoid	M2	16.12	no	Sigma, 62696	≥99
15	Quercetin	flavonoid	M2	16.35	no	Sigma, Q0125	≥98
16	Apigenin	flavonoid	M2	19.08	no	Sigma, 10798	≥95
17	Kaempferol	flavonoid	M2	19.77	no	Supelco, 96353	not specified
18	Galangin	flavonoid	M2	25.64	no	Supelco, 92342	not specified

[a]The chromatographic methods are described in the main part of the manuscript section 4.3. [b]Purity for allicin is estimated from ^{1}H-NMR spectral data.
